# The molecular pathophysiology of depression and the new therapeutics

**DOI:** 10.1002/mco2.156

**Published:** 2022-07-21

**Authors:** Haihua Tian, Zhenyu Hu, Jia Xu, Chuang Wang

**Affiliations:** ^1^ Ningbo Key Laboratory of Behavioral Neuroscience Ningbo University School of Medicine Ningbo Zhejiang China; ^2^ Zhejiang Provincial Key Laboratory of Pathophysiology School of Medicine Ningbo University Ningbo Zhejiang China; ^3^ Department of Physiology and Pharmacology Ningbo University School of Medicine Ningbo Zhejiang China; ^4^ Department of Laboratory Medicine Ningbo Kangning Hospital Ningbo Zhejiang China; ^5^ Department of Child Psychiatry Ningbo Kanning Hospital Ningbo Zhejiang China

**Keywords:** (R)‐ketamine, (S)‐ketamine, ketamine, major depressive disorder (MDD)

## Abstract

Major depressive disorder (MDD) is a highly prevalent and disabling disorder. Despite the many hypotheses proposed to understand the molecular pathophysiology of depression, it is still unclear. Current treatments for depression are inadequate for many individuals, because of limited effectiveness, delayed efficacy (usually two weeks), and side effects. Consequently, novel drugs with increased speed of action and effectiveness are required. Ketamine has shown to have rapid, reliable, and long‐lasting antidepressant effects in treatment‐resistant MDD patients and represent a breakthrough therapy for patients with MDD; however, concerns regarding its efficacy, potential misuse, and side effects remain. In this review, we aimed to summarize molecular mechanisms and pharmacological treatments for depression. We focused on the fast antidepressant treatment and clarified the safety, tolerability, and efficacy of ketamine and its metabolites for the MDD treatment, along with a review of the potential pharmacological mechanisms, research challenges, and future clinical prospects.

## Graphical abstract

This review aimed to clarify the safety, tolerability, and efficacy of ketamine and its metabolites for the treatment of major depressive disorder (MDD), along with a review of potential pharmacological mechanisms, research challenges, and future clinical prospects. Many novel hubba proteins and MDD‐risk proteins were found, indicating that the current pharmacological mechanisms were just the tip of the iceberg.

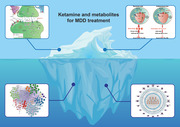

## 1 INTRODUCTION

Major depressive disorder (MDD) is a highly prevalent mental disorder and affects approximately 264 million patients worldwide, which makes it the second largest contributor to global morbidity^1^, ^2^. The World Health Organization (WHO) reports approximately 800,000 suicide cases per year^3^, which suggests that MDD is a significant public health challenge.

Mounting experimental and clinical studies have indicated that patients with depression have altered neuronal serotonergic and noradrenergic functions in the central nervous system (CNS).^4^, ^5^ Brain‐derived neurotropic factor (BDNF) may also play an important role in depression.^6^ Hypothalamic–pituitary–adrenal (HPA) axis hyperactivity is a common discovery in psychoneuroendocrinology studies of major depression.^7^ Furthermore, inflammatory cytokines and endogenous metabolites are involved in the mechanisms of depression,^8^ and additionally, the gut microbiome plays an critical role in depression by affecting the gut–brain axis.^9^

Presently, monoamine reuptake inhibitors are the most frequently prescribed class of antidepressants;^10^ however, there is a major issue with these due to the considerable lag time between the initial pharmacological effect on monoamine neurotransmitter function (up to several days) and the reduction in severity of clinical symptoms (usually a minimum of 3–8 weeks).^11^ Typically, after antidepressant drug therapy, only 50% of patients experience a major reduction in symptoms.^12^ Moreover, approximately 33% of all MDD cases involve treatment‐resistant depression (TRD), which is diagnosed when at least two courses of antidepressants have proven ineffective.^12^ Consequently, there is a need to identify and develop new, fast‐acting, and more effective antidepressant therapeutic agents.

Currently, many antidepressants are available, but their side effects make them less than ideal. This paper presents an overview of the molecular mechanisms that lead to depression, pharmacological treatment for depression, and the current status of ketamine as a treatment for depression. It can serve as a reference for studies on depression, as well as for the research for ideal rapid‐acting antidepressants.

## 2 PATHOLOGICAL MECHANISMS OF DEPRESSION

### 2.1 Hereditary

According to previous twin and adoption studies, major depression is likely to have a heritability rate of approximately 31–42%^13^ and may result from both genetic factors and the environment.^14^ The gene–environment interactions are believed to be crucial in explaining the etiology of major depression.^15^ However, there have been no strong and consistent genetic risk factors identified in genetic association studies due to the clinically heterogeneous nature of the disease and its complex genetic architecture.^16^ In light of this, identifying the individual genes responsible for depression has proven challenging. Nevertheless, several MDD risk loci have been identified.^17^, ^18^, ^19^ Studying a large cohort of MDD patients, Hyde et al. founded 15 genetic loci related to MDD risk in 2016.^20^ Recently, Wray et al. identified 44 risk loci via the largest genome‐wide association study (GWAS) meta‐analysis on MDD so far.^21^ Subsequently, Li et al. found three novel genetic loci related to the risk of MDD.^22^ In addition, a recent GWAS reported 102 independent variants linked with depression,^23^ and in another large cohort of individuals, these were linked to synaptic structures and neural transmission.^23^ This evidence suggests that MDD is influenced by genetic factors.

### 2.2 Neurotransmitter systems

Neurotransmitters are thought go play a critical role in depression etiology.^4^, ^24^ Serotonin (5‐HT) is widely distributed throughout the nervous systems and its deficiency can lead to depression, phobias, anxiety, and other mental health disorder in vertebrates.^25^ Over the past few decades, the 5‐HT hypothesis has driven research on the underlying cause of depression; with reports that depressed patients may have low brain 5‐HT levels and altered 5‐HT receptors, such as upregulated 5‐HT_2_ and downregulated 5‐HT_1A_ receptors.^26^ There are three possible mechanisms responsible for impaired 5‐HT_1A_ function in depression: social isolation reducing 5‐HT_1_ neurotransmission, 5‐HT_2_ receptors inhibiting 5‐HT_1_ neurotransmission, and hypercortisolaemia inhibiting 5‐HT_1_ neurotransmission.^27^ Endogenous proteins, such as BDNF and neurotrophin‐3, are related to the growth and function of 5‐HT neurons in the brains of adults.^28^

In the brain, dopamine (DA) is a dominant transmitter that regulates behavior and is a precursor to epinephrine and norepinephrine (NE).^29^ Numerous human and animal studies have shown that depression and DA transmission are closely related in the CNS.^30^, ^31^ Additionally, patients with depression have an increased level of DA transport,^32^ which makes presynaptic neurons more effective at reuptake DA.

Glutamate is the primary excitatory neurotransmitter and contributes to synaptic plasticity, cognitive activities, and motivational and emotional behavior in the brain.^33^ Multiple evidence suggests that depression is associated with the glutamate system.^34^, ^35^ Researchers have found elevated levels of glutamate in the blood, cerebrospinal fluid (CSF), and brains of patients with depression,^36^, ^37^ as well as N‐methyl‐D‐aspartate receptor (NMDAR) subunit disturbances in the brain.^38^, ^39^ The inhibition of NMDAR function has antidepressant effects and protects the hippocampal neurons from stress‐induced morphological changes.^40^ Furthermore, ketamine, an NMDAR antagonists, has been found to have rapid antidepressant effects.^41^ Alternatively, ketamine can enhance the α‐amino‐3‐hydroxy‐5‐methyl‐4‐isoxazolepropionic acid receptor (AMPAR) pathway by upregulating the AMPA glutamate receptor 1 subunit in hippocampal neurons.^42^ Additionally, antidepressants may also affect the AMPAR pathway.^43^

In contrast to glutamate, γ‐aminobutyric acid (GABA) is a primary inhibitory neurotransmitter. GABA neurons make up only a small percentage compared with glutamate neurons, but inhibitory neurotransmission is an important aspect of brain function because it balances excitatory transmission.^44^ GABA neurons are widely distributed in the brain and participate in many functions, including in the regulation of anxiety, motivation, and the reward system,^45^, ^46^, ^47^ and play an important part in alleviating the symptoms of MDD.^48^ Numerous studies have demonstrated that MDD patients have defects in GABA neurotransmission function.^49^, ^50^, ^51^ In a meta‐analysis of magnetic resonance spectroscopy studies, brain GABA levels in MDD patients were lower than those in healthy controls, but there was no difference seen between patients with depression in remission and healthy controls.^52^ A study by Mann et al. found that the level of GABA in the CSF of patients with MDD was lower than that in healthy controls.^53^ Several postmortem studies have demonstrated that low levels of GABA synthase and glutamic acid decarboxylase are present in the prefrontal cortex (PFC) of patients with depression.^54^, ^55^ There is evidence that depression is caused by an imbalance in the GABA and glutamate systems, and that GABA system activation produces antidepressant activity through the involvement of GABA_A_ receptor mediators a2/a3.^56^, ^57^ A GABA_A_ receptor mutant mouse model has shown that depression‐like behavior can be induced by altering the levels of potential GABA candidates in the brain.^58^, ^59^

### 2.3 Hypothalamic–pituitary–adrenal axis

Stress and acute challenges are factors contributing to MDD onset.^60^ It has long been recognized that the HPA axis plays an key role in mammals stress response. Therefore, changes in the HPA axis during depressive illness may reflect the influence of stress and determine the manifestations of depressive symptoms. Stress triggers the release of corticotropin‐releasing hormone (CRH) from the hypothalamus followed by stimulating adrenocorticotrophic hormone (ACTH) production in the pituitary, which subsequently increases glucocorticoids secretion from the adrenal cortex.^61^ The glucocorticoids interact with their receptors in multiple target tissues, such as the HPA axis, where they act as feedback inhibitors of both ACTH production in the pituitary corticotropes and CRH production in the hypothalamus.

In patients with depression, the HPA axis is overactive under stressful conditions, which results in problems such as hypercortisolemia, decreased rhythmicity, and elevated cortisol levels.^62^, ^63^ Disturbances of the HPA axis induced by stress have been shown to be associated with depression as a result of increased production of cortisol and insufficient inhibition of glucocorticoid receptor regulatory feedback.^64^, ^65^ Additionally, high cortisol levels have been linked to depression severity, particularly in cases of melancholic depression.^66^, ^67^ Moreover, patients with depression who could not normalize their HPA axis after treatment had a poorer clinical outcome and prognosis.^68^, ^69^ However, previous studies have shown that treatments that HPA axis‐regulating treatment like glucocorticoid receptor antagonists fail to alleviate the symptoms of depression.^70^, ^71^

### 2.4 Neurotrophins and neurogenesis

The findings of volumetric reductions in the hippocampus and other forebrain regions in depressed patients support the popular hypothesis that decrements in neurotrophic factors that regulate plasticity within the adult brain contribute to depression. The focus of these studies has largely been on BDNF, which plays important roles in different aspects of the nervous system, including synaptic plasticity, differentiation, maintenance, neuronal outgrowth, and repair.^72^ The neurotrophin hypothesis of depression is primarily based on the theory that reduced hippocampal BDNF levels are associated with stress‐induced depression and are elevated by treatment with antidepressants.^73^, ^74^ Agents targeting the BDNF system have been found to produce antidepressant‐like effects.^75^, ^76^ Moreover, mounting research shows that BDNF levels are reduced in the postmortem peripheral blood of patients with depression,^77^, ^78^, ^79^, ^80^, ^81^ and some reports have indicated that antidepressant treatment can normalize this.^82^, ^83^ Additionally, there is evidence that the interaction between BDNF and its receptor is related to TRD.^84^.It appears that BDNF depletion impairs neurogenesis and contributes to the onset of MDD, and the antidepressant can mitigate MDD symptoms by increasing BDNF levels in the brain.

### 2.5 Neuroinflammation

Some psychiatric studies over the past two decades have hypothesized that inflammation is linked to the pathogenesis and pathophysiology of major depression. Numerous early studies have found depression to be more common in patients who had autoimmune or infectious diseases than in the general population.^85^ Moreover, even individuals who do not suffer from depression may exhibit depressive symptoms when exposure to cytokines, while antidepressants ease this discomfort.^86^

MDD patients have been demonstrated to have increased levels of inflammatory molecules,^87^, ^88^, ^89^ and display hallmarks of immune‐inflammatory response through evidence of elevated proinflammatory cytokines and their receptors, chemokines, and soluble adhesion molecules in their peripheral blood and CSF.^90^, ^91^, ^92^ Peripheral inflammatory markers not only affect the state of immune activation in the CNS, which, in turn, impacts explicit behavior, but can also serve as evaluation or biological indices for antidepressant therapy.^93^, ^94^ Li et al. showed that tumor necrosis factor alpha (TNF‐α) levels in MDD patients were higher before treatment than those in healthy controls. When treated with venlafaxine, TNF‐α levels decreased significantly. Furthermore, there was a greater decrease in TNF‐α levels in the group of patients for whom the treatment was effective.^95^ Antidepressants significantly reduced the level of peripheral interleukin‐6 (IL‐6), TNF‐α, IL‐10, and the C‐C motif ligand 2 chemokine, suggesting that antidepressants may reduce the markers of peripheral inflammation.^96^ Moreover, Syed et al. found that untreated depression patients inflammatory markers were higher, and when they were treated with antidepressants, the levels of anti‐inflammatory cytokines increased; whereas in nonresponders, there was an increase in proinflammatory cytokines.^94^ Various studies have also suggested that cytokine inhibitors like monoclonal antibodies may exert an antidepressant effect by blocking cytokines.^97^ An imbalance between proinflammatory and anti‐inflammatory cytokines may contribute to the pathophysiology of depression.

Microglia are known to contribute to neuronal plasticity and play a role in MDD development.^98^, ^99^ A study by Weng et al. found a higher number of microglia in the PFC of mice intraperitoneally injected with lipopolysaccharide (LPS), whereas mouse depressive behavior also increased.^100^ These researchers also observed the upregulation of IL‐1, IL‐6, and TNF‐α gene expression in the mouse PFC, which was suppressed by selective 5‐HT reuptake inhibitor (SSRI). Additionally, astrocytes have been implicated in the pathogenesis of stress‐ and LPS‐induced inflammation caused by depressive symptoms.^101^ As activated microglia cause inflammation through excessive levels of proinflammatory factors and cytotoxins, depression‐like behavior may gradually develop.^98^, ^102^

### 2.6 Metabolic disorders

Patients with MDD often suffer from metabolic disorders, and those with metabolic disorders are inclined to experience depression.^103^, ^104^, ^105^ The development of effective analytical technologies and methods for the analysis of fluids and tissues from a diseased organism allows us to gain a greater understanding of the basis for diseases.^106^ Experimental findings in animal models and clinical practice indicate that metabolomics can be used to investigate the pathophysiology of depression and potential biomarkers.

Metabolomics has been shown to be an effective tool for selecting appropriated animal models to study depression.^107^ Zheng et al. found 23 differentially expressed metabolites that distinguished MDD subjects from healthy control subjects, and identified five metabolites as potential biomarkers that can be used to differentiate MDD subjects accurately.^108^ The key metabolites included amino acids and lipid/protein complexes, and some molecules related to lipid metabolism and energy metabolism that contributed to the discrimination between depressed patients and healthy controls were also identified.^109^

To identify depression‐related biomarkers, gas chromatograph‐mass spectrometry (GC‐MS) was applied to metabolomic analysis of plasma samples collected from chronic unpredictable mild stress (CUMS‐induced) rats. Li et al. reported that 12 metabolites concentrations in the CUMS group were significantly different from those in the control group. The Kyoto Encyclopedia of Genes and Genomes (KEGG) pathway database revealed that CUMS treatment affected amino acid metabolism, energy metabolism, and glucometabolism.^110^ Using GC‐MS‐based metabolomics, CUMS rats had lower levels of isoleucine and glycerol, whereas N‐acetylaspartate and β‐alanine levels were higher than those in control rats.^111^ Furthermore, Gao et al. found that six potential biomarkers (glycine, glutamate, fructose, citric acid, glucose, and hexadecenoic acid) are closely associated with depression.^112^

### 2.7 Microbiome–gut–brain axis

Recent attention has been drawn to the microbiota–gut–brain axis owing to its potential to regulate brain activity. Several studies have revealed that the microbiota–gut–brain axis is important for regulating mood, behavior, and neuronal transmission in the brain,^113^, ^114^ and is associated with MDD.^115^, ^116^, ^117^ Several studies have suggested that depression and gastrointestinal disorders are comorbid.^118^, ^119^ Some antidepressants can alleviate the symptoms of people suffering from irritable bowel syndrome and other related disorders.^120^ In patients with MDD, alterations in the gut microbiome have been reported,^121^, ^122^ and related to depression‐like behaviors^123^, ^124^ and brain function.^125^ Studies on animals have demonstrated that stress can alter the composition and diversity of the intestinal microflora, and that this is accompanied by depressive behavior.^126^, ^127^ It is interesting to note that rodents display depressive behavior following fecal transplantation from human patients with depression.^124^ In contrast, some probiotics have been found to ameliorate depression‐like behavior in preclinical studies^128^ and to have antidepressant effects in several double‐blind, placebo‐controlled clinical trials involving patients with depression.^129^, ^130^

Gut microbiota can influence the brain in several ways, such as the HPA axis and the neuroendocrine‐, autonomic‐, and neuroimmune systems.^131^ Recent studies have demonstrated that the gut microbiota can influence the levels of certain neurotransmitters, including 5‐HT, DA, noradrenalin, glutamate, and GABA in the gut and brain.^132^ Additionally, recent studies have indicated that changes in the gut microbiota can damage the gut barrier and increase peripheral inflammatory cytokines.^133^, ^134^ Furthermore, short‐chain fatty acids, such as butyrate, are known to increase BDNF levels, whereas gut dysbiosis decreases BDNF levels; which could have an impact on neuronal development and synaptic plasticity.^115^ There has been significant progress in research in this area, but more clinical trials are required to determine whether probiotics are effective in treating depression. In addition, the underlying mechanisms need to be elucidated.

### 2.8 Other systems and pathways

It is clear that a number of additional systems or pathways are also thought to play a role in the pathophysiology of depression, such as oxidant‐antioxidant imbalance,^135^ mitochondrial dysfunction,^136^, ^137^ and circadian rhythm‐related genes;^138^ especially their critical interactions (e.g., interactions between the HPA and mitochondrial metabolism^139^, ^140^), and the reciprocal interaction between oxidative stress and inflammation.^135^

**FIGURE 1:**
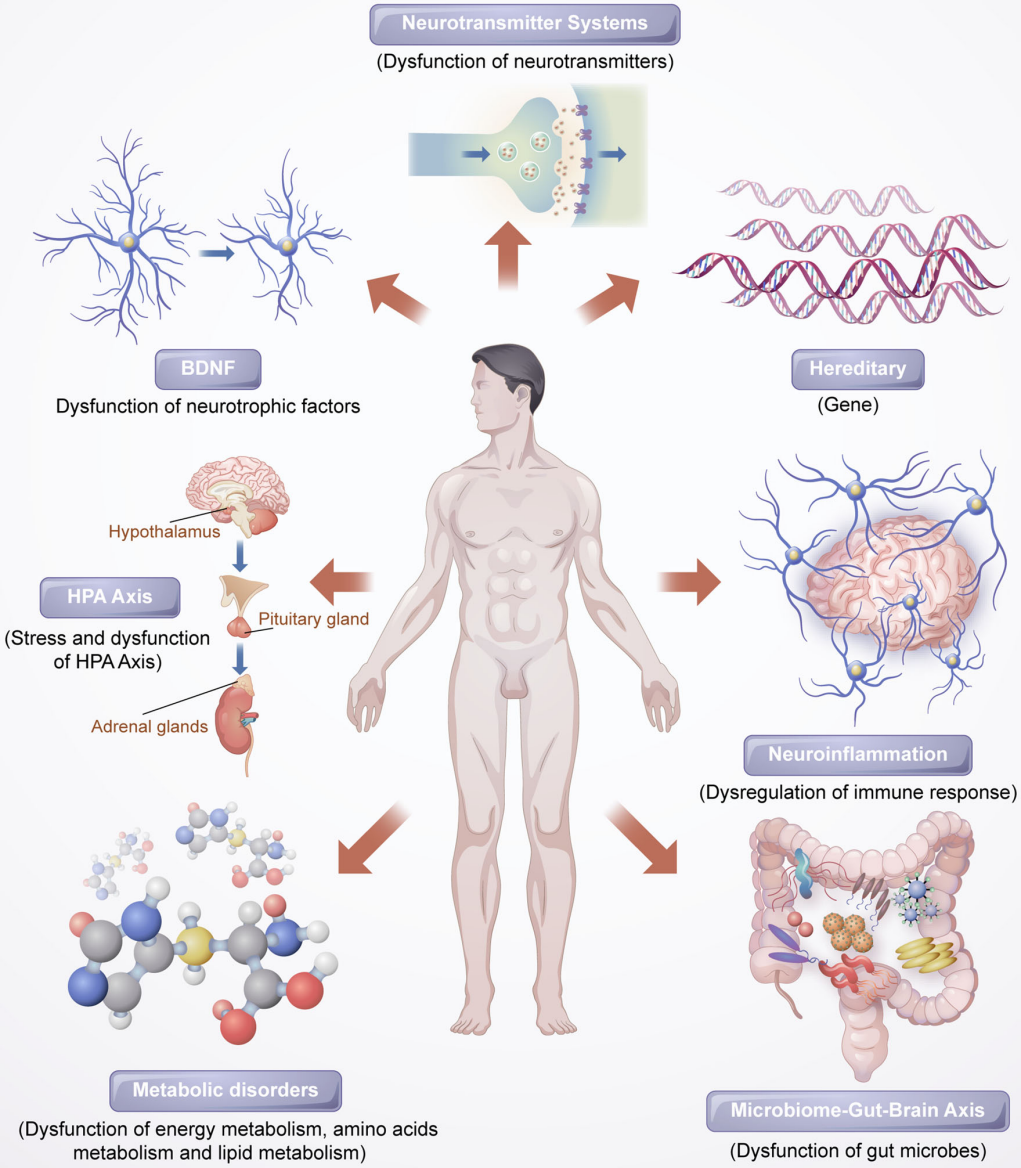
The potential underlying mechanisms of depression

We still do not fully understand the causes of depressive disorders in spite of the abundance of research on the disease and numerous hypotheses nowadays. Different researchers have performed a variety of tasks related to modality from linked and complementary perspectives, which is helpful to further our understanding of depression. A comprehensive understanding of depression pathogenesis should consider interactions between various systems and pathways. Figure 1 shows the various pathological mechanisms of depression.

## 3 PHARMACOLOGICAL TREATMENT OF DEPRESSION

Antidepressant medications are commonly prescribed for the treatment of MDD, including tricyclic antidepressants (TCAs), monoamine oxidase inhibitors (MAOIs), selective serotonin reuptake inhibitors (SSRIs), serotonin noradrenaline reuptake inhibitors (SNRIs), serotonin‐2 antagonists and reuptake inhibitors (SARIs), NE reuptake inhibitors (NDRIs), specific serotonergic antidepressants (NaSSAs), and multimodal antidepressants (MMAs). Most antidepressant drugs primarily affect the brain. Monoamine neurotransmitters, 5‐HT, NE, and DA, although antidepressant drugs differ in their selectivity.

### 3.1 TCAs

TCAs were developed to improve the mood of depressed patients in the 1950s.^141^ TCAs inhibit the neuronal uptake of NE and 5‐HT.^142^, ^143^ The treatment response to TCAs results in a higher availability of 5‐HT or NE at postsynaptic receptor sites. Even though TCAs have increasingly been replaced by SSRIs and other new safer antidepressants, they remain an excellent option for some patients whose depression does not improve after treatment with less potent antidepressants. Among TCAs, the antagonistic effects of adrenergic, muscarinic, and histaminergic receptors are responsible mainly for the side effects of dizziness, memory impairments, and drowsiness.^142^, ^143^, ^144^

### 3.2 MAOI_S_

MAOIs were among the first antidepressants to be licensed for managing depression. MAOIs inhibit the monoamine oxidase enzyme, thus demonstrating that depression is a neurochemical disorder that can be managed with medication that correct underlying neurotransmitter imbalances.^145^ MAOIs are rarely prescribed today, or they are the last antidepressants used because of hypertensive crisis caused by severe and potentially fatal food and drug interactions.^146^

### 3.3 SSRIs

SSRIs, known as fluoxetine, sertraline, citalopram, and paroxetine, are the most commonly type of antidepressants around the world to treat depression and are prescribed as the first‐line treatment. SSRIs work by decreasing the reuptake of 5‐HT, so that more of it remains at the receptor sites to alleviate mood.^147^ Nowadays, people suffering adverse reactions to one SSRI may find it helpful to switch to another drug from this group. SSRIs are safer than MAOIs and TCAs, have fewer side effects, and are less likely to cause death from overdose owing to the lack of affinity for acetylcholine receptors and amines receptors.^146^ Nevertheless, many side effects, including nausea, insomnia, and sexual dysfunction, are caused by SSRIs.^148^

### 3.4 SNRIs

SNRIs, such as venlafaxine and duloxetine, work in the same way as TCAs as they inhibit the 5‐HT and NE reuptake at the respective transporters. Nevertheless, SNRIs have little or no pharmacological action at adrenergic (α_1_, α_2_, and β), histamine (H_1_), muscarinic, DA, or postsynaptic 5‐HT receptors, differently from TCAs.^149^, ^150^, ^151^, ^152^, ^153^ According to some reports, SNRIs may be more effective at treating MDD than SSRIs. Comparatively, these differences are modest.^154^, ^155^ SNRIs have comparable clinical tolerability and a similar prevalence of sexual dysfunction compared with other antidepressant drug treatments.^155^, ^156^

### 3.5 SARIs

SARIs such as trazodone and its analog nefazodone are antidepressants that have the ability to inhibit the reuptake of 5‐HT and NE, interact with α_1‐_adrenoceptors, and have no effect on histaminergic or cholinergic receptors.^157^ SARIs have comparative efficacy and lower rate of induced sexual dysfunction compared to other classes of antidepressant for treatment of MDDs.^158^

### 3.6 NDRIs

The NDRI bupropion is the only antidepressant that has a dual action on NE and DA neurotransmitter systems, which is very different from other antidepressant drugs (i.e., TCAs, SSRI, and SNRI). Bupropion has the strongest binding affinity for DA transporters compared to that of NE transporters and minimal or no binding affinity for 5‐HT transporters or other pre‐ and postsynaptic receptors.^152^, ^159^, ^160^, ^161^, ^162^ Clinical research has demonstrated that bupropion is as effective as other antidepressant drugs for treating MDD. Bupropion is well tolerated, and its most frequent side effects are dry mouth, nausea, and insomnia.^163^, ^164^, ^165^ Additionally, bupropion has the lowest incidence of sexual dysfunction compared to that of TCAs, MAOIs, SSRIs, and SNRIs.^156^, ^166^

### 3.7 NaSSA

Mirtazapine (Remeron, Zispin) is the NaSSA class approved in many counties for its use in the treatment of major depression.^167^ Mirtazapine increases central noradrenergic and serotonergic activity by blocking α_2_ adrenoceptors and selectively inhibiting 5‐HT_2_ and 5‐HT_3_ receptors.^167^, ^168^ Mirtazapine appears to exert its effect faster than antidepressant reuptake inhibitors during the acute treatment phase of major depression.^169^, ^170^ The main side effect of mirtazapine is weight gain, which seems to occur earlier in the course of the treatment, and becomes less of an issue as the treatment continues.^171^

### 3.8 MMAs

Vortioxetine as well as vilazodone belong to the chemical class of the piperazines and is a new class of antidepressant drugs called MMA agents because vortioxetine exhibits high binding affinity for multiple 5‐HT receptors (such as 5‐HT_1A_, 5‐HT_1B_, 5‐HT_3A_, 5‐HT_7_, and 5‐HTtransporters).^172^, ^173^, ^174^ Thus, vortioxetine may affect the activity of various neurotransmitter systems, including 5‐HT, NE, DA, acetylcholine, histamine, glutamate, and GABA.^175^ Vortioxetine is comparable to other antidepressants in terms of its clinical efficacy and tolerability, with nausea and headaches being the most common side effects. Vortioxetine appears to be associated with low risk of sexual dysfunction and weight gain.^176^, ^177^, ^178^, ^179^ Vortioxetine has been shown to enhance cognitive functioning through its action at 5‐HT_3_ and 5‐HT_1A_ receptors in clinical and preclinical studies.^174^, ^180^, ^181^, ^182^

Although there are several classes of antidepressant drug, the benefits of current available treatments for depression are limited due to the low response rates, delayed therapeutic effects, and multiple side effects. Their long therapeutic delays (up to 3 weeks) and low rates of remission (approximately 30%) have prompted the search for more effective therapies.^183^ Therefore, A more effective, faster‐acting, and nonmonoaminergic‐based antidepressant medication is urgently needed. The noncompetitive NMDA receptor antagonist ketamine, which has consistently been proven to produce rapid and sustained antidepressant effects and alleviated suicidal ideation in MDD patients in multiple clinical studies,^184^, ^185^ has shown to be the most promising novel glutamatergic‐based treatment for MDD.

## 4 DISCOVERY OF THE FAST‐ACTING ANTIDEPRESSANT ACTIONS OF KETAMINE

### 4.1 The discovery of ketamine as an antidepressant

**FIGURE 2:**
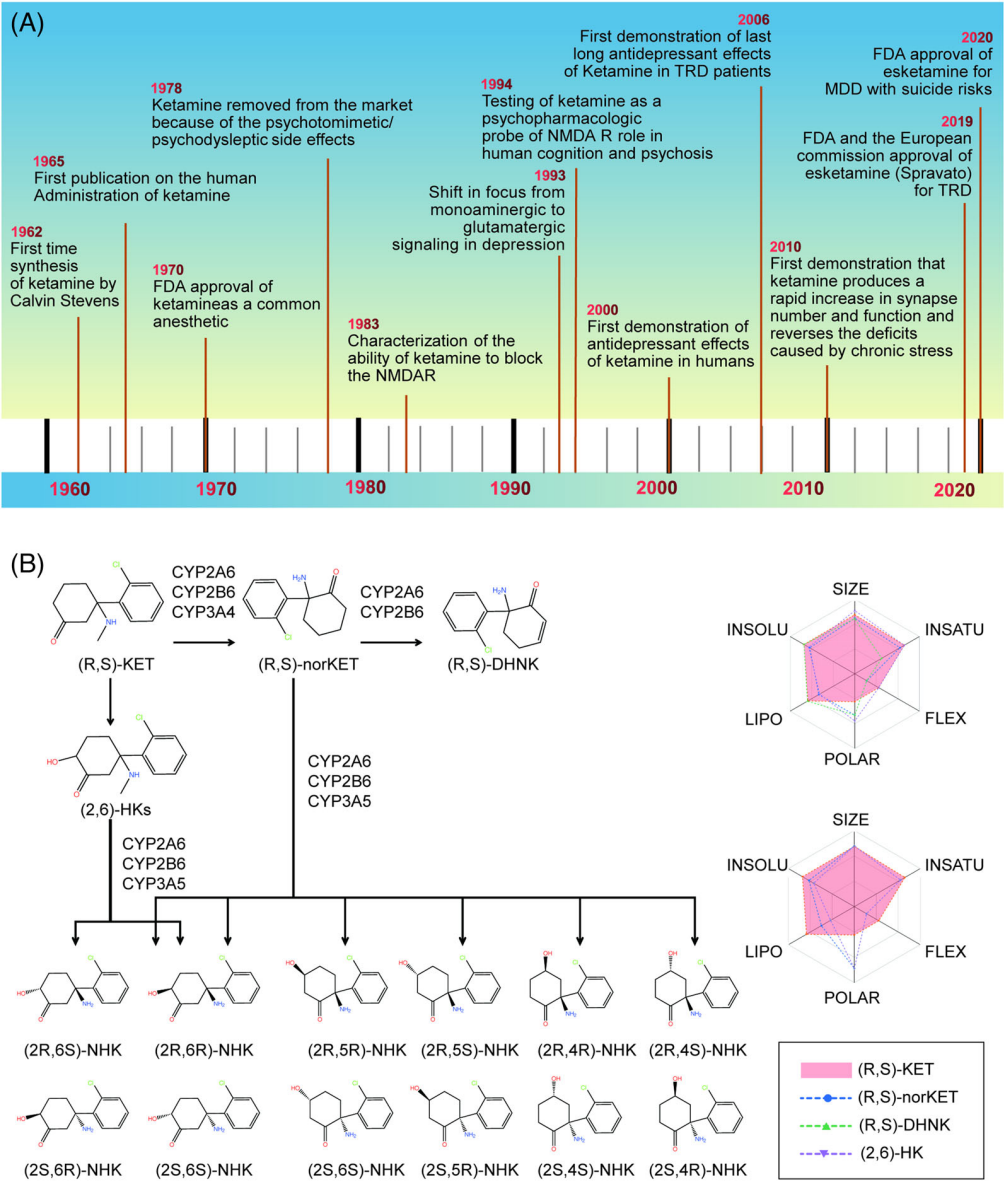
Timeline of the key events history of ketamine and its metabolic formation of hydroxynorketamines from ketamine. (A) The key events of the ketamine findings and development for MDD. (B) The ADME parameters, pharmacokinetic properties, drug‐like nature, and medicinal chemistry friendliness of ketamine metabolites predicted by SwissADME. The radar plot reflects the physicochemical properties in regard to six aspects: SIZE (molecular weight), INSOLU (solubility), LIPO (lipophilicity), POLAR (polarity), FLEX (flexibility), and INSATU (insaturation). The structure formula of Figure 2B was drawn by ChemDraw20.0.

Treatment failures and delays in clinical improvement with traditional antidepressants, developed based on the monoamine hypothesis of depression, prompted the discovery and development of antidepressants using multiple target discovery strategies. As shown in Table 1, we list antidepressants approved between 2000 and 2021. (*R,S*)‐ketamine (hereafter referred to as ketamine) is a phenylcyclohexylamine derivative (mol.wt. = 237.73) initially characterized by Lodge et al.^186^ as an NMDARs antagonist (Ki = 0.53 μM for NMDARs), which supported the glutamate hypothesis of depression and its implications for antidepressant treatments. Evidence that glutamatergic agents might have antidepressant efficacy dates back as far as 60 years ago.^187^ Recently, the revolutionary discovery and approval of the fast‐acting antidepressant ketamine has marked a landmark in the field of psychiatry in the past half century. However, the development of ketamine's rapid and sustained antidepressant effects for the treatment of MDD has experienced a tortuous process that has led to new insights into novel antidepressants (as shown in Figure 2A). The first publication on the administration of ketamine in humans was reported in 1965;^188^ subsequently, ketamine became commercially available for human consumption in 1970,^189^ and was widely utilized as an intravenous anesthetic drug. However, ketamine was subsequently taken off the market in 1978 because of its psychotomimetic/psychodysleptic side effects.^190^

**TABLE 1:** Antidepressants approved from 2000 to 2021

Effective constituent	Mechanisms of action	Adaptation disease	Listing country and time
Aripiprazole	Partial agonist of D2 receptor Partial agonist of 5‐H1A receptor Partial agonist of 5‐HT2A receptor	Adjuvant treatment of MDD	American (2002) European (2004) Japan (2006) China (2006)
Escitalopram	SSRI	MDD	American (2002) European (2001) Japan (2011) China (2005)
Duloxetine	SNRI	MDD	American (2004) European (2004) Japan (2010) China (2006)
Quetiapine	Partial agonist of D2 receptor Partial agonist of 5‐HT2A receptor	Adjuvant treatment of MDD	American (2007) European (2010) Japan (2012) China (2008)
Agomelatine	Melatonin	Depression	European (2009) China (2011)
Desmethylvenlafaxine	SNRI	Depression	American (2008)
Vilazodone	SNRI Partial agonist of 5‐H1A receptor	MDD	American (2011)
Levomilnacipran	SNRI	MDD	American (2013)
Vortioxetine	SNRI Antagonist of 5‐HT3,5‐HT7,5‐HT1A receptor	MDD	American (2013) European (2013) China (2017)
Brexpiprazole	Partial agonist of D2 receptor Partial agonist of 5‐HT1A receptor Antagonist of 5‐HT2A receptor	Adjuvant treatment of MDD	American (2015) Japan (2018) European (2018)
Esketamine	Antagonist of NMDAR	TRD	American (2019) European (2019)
Brexanolone	GABA_A_ receptor modulator	Postpartum depression	American (2019)

GABA, γ‐aminobutyric acid; 5‐HT1A, Serotonin 1A; 5‐HT2A, Serotonin 1A; 5‐HT3, Serotonin 3; 5‐HT7, Serotonin 7; 5‐HT2A, Serotonin 2A; MDD, major depressive disorder; NMDAR ,N‐methyl‐d‐aspartate receptor; SNRI, serotonin and norepinephrine reuptake inhibitor; SSRI, selective serotonin reuptake inhibitor; TRD, treatment‐resistant depression.

Drug repurposing of ketamine found that its antidepressant actions had been identified in a 1975 preclinical study,^191^ and a clinical study was conducted in 1973 by Khorramzadeh and Lotfy.^192^ Notably, the first placebo‐controlled study in humans showing ketamine's fast antidepressant actions was reported in 2000^193^ and its long‐lasting antidepressant effects were reported in 2006.^194^ Ketamine was once again the subject of interest in antidepressant due to reports that subanesthetic levels of the drug (0.5 mg/kg), administered intravenously (i.v.), generated a rapid and sustained antidepressant function in patients with MDD.^193^ Subsequently, Zarate and colleagues corroborated this finding with their own double‐blind, placebo‐controlled, crossover investigation of unipolar/bipolar depression cases.^194^, ^195^ Following this seminal study, multiple studies have validated the rapid action of ketamine in the treatment of MDD/TRD/unipolar/bipolar depression.^193^, ^194^, ^196^, ^197^ Emerging evidence demonstrates that the antidepressant effect of ketamine occurs within hours of its administration and typically lasts for days to weeks depending on individual cases.^185^, ^194^, ^198^ Most notably, this rapid action is remarkably effective against suicidal thoughts. Randomized controlled trials (RCTs) have confirmed a reliable ketamine‐driven antidepressant function in TRD cases.^194^, ^196^, ^199^, ^200^, ^201^, ^202^, ^203^, ^204^, ^205^ Notwithstanding, the antidepressant functions provided by ketamine are transient, with a typical time frame being 7 days following one infusion dose,^194^, ^196^, ^199^, ^200^ and approximately 18–19 days after multiple infusion doses.^197^, ^206^ Retrospective data from real‐world studies find that true‐life medical information exhibited 44% responsive rates following six i.v. ketamine doses in cases of TRD with multiple comorbidities.^207^ Moreover, ketamine has exhibited antisuicide functions and anti‐anhedonic effects.^184^, ^208^, ^209^, ^210^, ^211^ Moreover, its pharmacology is different from that of well‐established antidepressant drugs and consequently deemed to be a major advancement in mood‐disorder therapeutics over the preceding 60 years.^212^ Importantly, an enantiomer of racemic ketamine, (*S*)‐ketamine, was shown to be effective in an antidepressant clinical trial,^202^, ^203^, ^204^, ^205^ and to decrease suicidal ideation in patients suffering from depression.^213^. Although four of the five esketamine ((*S*)‐isomer) phase 3 clinical trials found no difference with placebo,^214^, ^215^ the United States Food and Drug Administration (FDA) decided to grant approval based on the positive results of one trial and promising data from one of the other trials. Esketamine intranasal spray *(Spravato)* was approved by the United States FDA in March 2019 as an adjunctive treatment for TRD cases of MDD in a supervised setting,^216^, ^217^ and in 2020 for patients with MDD and suicidal risks(https://www.fda.gov/news‐events/press‐announcements/fda‐approves‐new‐nasa‐spray‐medication‐treatment‐resistant‐depression‐available‐only‐certified).

### 4.2 The composition and metabolic form of ketamine

Ketamine is a racemic mixture of equal amounts of (*S*)‐ketamine and (*R*)‐ketamine. Following demethylation of ketamine to norketamine, norketamine is further metabolized to hydroxynorketamines (HNKs) and dehydronorketamine (DHNK). As shown in Figure 2B, the metabolic formation of HNKs from ketamine and the absorption, distribution, metabolism, and excretion (ADME) parameters, pharmacokinetic properties, drug‐like nature, and medicinal chemistry friendliness of ketamine metabolites were predicted by SwissADME. (*S*)‐Norketamine is a metabolite of (*S*)‐ketamine formed by the cytochrome P450(CYP) enzymatic complex. A preclinical study showed that, similar to (*S*)‐ketamine, (*S*)‐norketamine produced antidepressant actions in different rodent depression models,^218^, ^219^ and in contrast to ketamine and (*S*) ketamine did not produce detrimental side effects such as the risk of misuse/abuse, prepulse inhibition (PPI) issues, exacerbated baseline γ‐oscillations, or a decrease in parvalbumin (PV) immunoreactivity within the medial PFC (mPFC) in mice.^218^, ^220^, ^221^ In such in vivo models, (*S*)‐norketamine was found to be equivalent in potency to (*S*)‐ketamine in terms of antidepressant‐type activities, although it had reduced potency in comparison to (*R*)‐ketamine. Therefore, (*S*)‐norketamine appears to have a higher safety profile than the parent compound (*S*)‐ketamine for human antidepressant function^218^, ^219^, ^220^ and is thus a safer alternative. However, recent findings on the superiority of (*R*)‐ketamine versus (*S*)‐ketamine in animal models of depression are in agreement with previous studies by Hashimoto et al., which revealed that (*R*)‐ketamine has shown to have greater potency and long‐term antidepressant actions than (*S*)‐ketamine in several rodent models of depression.^222^, ^223^ (*2R,6R*)‐HNK, the main metabolite of (*R*)‐ketamine, plays a critical role in the rapid‐acting effects of ketamine.^224^, ^225^, ^226^ Importantly, it has attracted especially high interest as a candidate antidepressant in its own right.^226^ (*2R,6R*)‐HNK was found to induce antidepressant‐type functions with no adverse events in rodents.^227^ Nevertheless, recent preclinical studies found that (*2R,6R*)‐HNK had no rapid or sustained antidepressant effects in animal studies.^228^, ^229^, ^230^ Currently, there is still an considerable debate regarding the antidepressant effects of (*2R,6R*)‐HNK. Even so, a phase I clinical trial for (*R*)‐ketamine and (2*R*, 6*R*)‐HNK was initiated in early 2019.^231^ It would be interesting to directly compare (*R*)‐ketamine, *(S)*‐ketamine, and (2*R*, 6*R*)‐HNK in patients with depression.

Ketamine has been clinically used as an anesthetic since the 1970s. However, indications for its use as an antidepressant and the molecular mechanisms of its metabolites are still emerging. Several molecular and cellular targets have been identified, yet the pharmacokinetics, pharmacodynamics, candidate targets, and side effects should be investigated further in order to understand the detail neurobiological mechanisms underlying the effects of ketamine and its metabolites. As shown in the Table 2, we summary the published clinical studies of intravenous ketamine and intranasal esketamine in depressive patients.

**TABLE 2:** The clinical study for the antidepressant effects of ketamine

Study design	Diagnosis/patients	Sample size	Ketamine or metabolitesadministration	Other medications	Comparator	Key outcome measures/Instrument	Result/conclusions	Reference
Randomized, Double‐blind, placebo‐controlled	Unipolar or bipolar depression	9	0 or 0.5 mg/kg over 40 min i.v. (single dose of ketamine)	Subjects were drug free	Saline	HAMD‐25	Significant improvements in depressive symptoms within 72 h after ketamine but not placebo infusion.	^193^
Randomized, Double‐blind, placebo‐controlled Crossover	MDD	18	0 or 0.5 mg/kg over 40 min i.v. (single dose of ketamine)	Subjects were drug free	Saline	HAMD‐21	Of subjects treated with ketamine, 71% responded after 1 day, and 35% maintained a response for ≥1 week.	^194^
Randomized, placebo‐controlled continuation	Unipolar TRD	26	0.5 mg/kg over 40 min i.v. (open‐label, single dose of ketamine)	2 h before ketamine: Randomized, lamotrigine or placebo 72 h after ketamine: Responders randomized, riluzole or placebo		MADRS	Responses were observed in 65% of subjects at 24 h and 54% of subjects at 72 h. Lamotrigine did not attenuate the mild, transient side effects of ketamine, and did not enhance its antidepressant effects. Riluzole did not prevent relapse in the first month after ketamine treatment.	^185^
Randomized, Double‐blind, placebo‐controlled	Unipolar TRD	42	0.5 mg/kg over 40 min i.v. (open‐label, single dose of ketamine)	Randomized, placebo or riluzole, starting after ketamine infusion		MADRS	At 4–6 h after the ketamine infusion, 62% of subject had responded. The average time to relapse was approximately 17.2 days in the ketamine‐riluzole group and 9.8 days in the ketamine‐placebo group.	^201^
Multiple dose, open‐label, three times weekly over 12 days	TRD	24	0.5 mg/kg over 40 min i.v. of ketamine	Subjects were drug free		MADRS	70.8% of subjects were responders; response was sustained for the duration of the study. Median time to relapse in responders was 18 days.	^197^
Double‐blind, randomized,	MDD	73	0.5 mg/kg 40 min infusion of ketamine		Midazolam	MADRS	The likelihood of response at 24 h was greater with ketamine than with midazolam with response rates of 64% and 28%, respectively.	^196^
Randomized, double‐blind, crossover study	MDD,TRD, 21–65 years	18	50 mg of racemic ketamine(once per week)		0.9% saline solution	MADRS	8 of 18 patients (44%) 24 h after ketamine administration compared with 1 of 18 (6%) after placebo	^232^
Double‐blind, multicenter, proof‐of‐concept study	MDD,19‐64 years	68	84 mg of esketamine (56 mg if intolerance) twice weekly for 4 weeks		Placebo	MADRS	Change from baseline in MADRS total score to 4 h, 24 h, and 25 day	^213^
Randomized, multicenter, double‐blind, and active‐controlled; fixed dosing	Adults with TRD; age group = 18–64	346	Esketamine 56 mg or 84 mg given intranasally two times per week for 4 weeks	Subjects were treated with OAD (duloxetine, escitalopram, sertraline, or venlafaxine)	Placebo plus OAD	MADRS; CGI‐S; SDS; PHQ‐9; GAD‐7; EQ‐5D5L; CADSS; BPRS; MOAA/S; GADR; PWC	No statistically significant difference was seen between treatment with ESK plus OAD group compared to placebo plus OAD group	^215^
Randomized, multicenter, double‐blind, and active‐controlled; flexible dosing	Adults with TRD; age group = 18–64	223	Esketamine 56 mg or 84 mg given intranasally two times per week for 4 weeks	Subjects were treated with OAD duloxetine, escitalopram, sertraline, or venlafaxine)	Placebo plus OAD	MADRS; SDS; PHQ‐9; GAD‐7; EQ‐5D‐5L; CGIS; C‐SSRS; CADSS; BPRS; MOAA/S; CGADR; PWC	Treatment with ESK plus OAD was associated with a significantly greater change in MADRS score compared to placebo plus OAD	^233^
Randomized, multicenter, double‐blind, and active‐controlled; flexible dosing	Adults with TRD; age group ≥ 65 years	138	Esketamine 28 mg or 56 mg or 84 mg given intranasally two times per week for 4 weeks	Subjects were treated with OAD (duloxetine, escitalopram, sertraline, or venlafaxine, daily for 4 weeks)	Placebo plus OAD	MADRS; CGI‐S; PHQ‐9; SDS; CSSRS; CADSS; BPRS; CSCB; HVLT‐R; MOAA/S; CGADR; PWC	No statistically significant difference was seen between treatment with ESK plus OAD Group compared to placebo plus OAD group	^202^
Randomized withdrawal design, double‐blind, multicenter, active controlled	Adults with TRD; age group = 18–64	705	56 mg or 84 mg intranasally twice a week of esketamine	OAD were used		MADRS used, and the relapse time was assessed between the two treatment groups	Significantly delayed relapse of depressive symptoms observed in esketamine plus OAD group	^203^
Long‐term (one year) study, multicenter, Open‐label; phase 3	Adults with TRD; ≥18 years	802	Esketamine 28 mg (for ≥65 years), 56 or 84 mg given intranasally twice weekly during the 4‐week induction phase (given along with OAD)	OAD (duloxetine, escitalopram, sertraline, or venlafaxine) were used		MADRS; CSCB; DET; IDN; OCL; ONB; GMLT; HVLT‐R; CSSRS; CADSS; BPRS; MOAA/S; BPIC‐SS; PWC; PHQ‐9; SDS; CGI‐S	Improvement in depressive symptoms was found to be sustained in patients with TRD	^204^
Double‐blind, phase 3 studies	MDD with acute suicidal ideation or behavior	456	Esketamine 84 mg or placebo nasal spray twice weekly for 4 weeks	Comprehensive standard of care, including hospitalization and newly initiated or optimized antidepressants		MADRS scale and clinical global impression severity of suicidality‐revised were used to evaluate changes from baselines at 24 h after the first dose	Esketamine plus comprehensive standard of care rapidly reduces depressive symptoms in patients with major depressive disorder who have acute suicidal ideation or behavior	^205^

HDRS, Hamilton Depression Rating Scale; MADRS, Montgomery–Asberg Depression Rating Scale; TRD, treatment‐resistant depression; OAD, oral antidepressant; MDD, Major depressive disorder; IV, Intravenous; IN, Intranasal; mg, Milligrams; kg, Kilograms; SD, standard deviation; SE, Standard error; CI, confidence interval; LSMD, least square mean difference; AD, antidepressants; BPIC‐SS, bladder pain‐interstitial cystitis symptoms scale; BPRS, brief psychiatric rating scale; CADSS, clinician‐administered, dissociative states scale; CGADR, clinical global assessment of discharge readiness; CGI‐S, clinical global impression severity; CGI‐I, clinical global impression improvement; CSCB, Cog state computerized battery; C‐SSRS, Columbia suicide severity rating scale; EQ‐5D‐5L, EuroQol‐5 dimension‐5 level; DET, detection task; EWPS, Endicott work productivity scale; GAD‐7, generalized anxiety disorder 7‐item; GADR, global assessment of discharge readiness; GMLT, Groton maze learning test; HAM‐A, Hamilton anxiety rating scale; HVLT‐R, Hopkins verbal learning test‐revised; IDN, identification task; LFT, liver function tests; MOAA/S, modified observer's assessment of alertness/sedation; OCL, one card learning; ONB, one back; PHQ‐9, patient health questionnaire 9‐item; PRISE, patient‐rated inventory of side effects; PWC, physician withdrawal checklist; QIDS‐SR16, quick inventory for depressive symptomatology self‐report 16‐item; SDS, Sheehan disability scale; SF‐12, short form health survey; SHAPS, Snaith–Hamilton pleasure scale; YMRS, Young mania rating scale; 2 BACK, two back task; CADSS, the clinician administered dissociative states scale.

## 5 EFFICACY AND SAFETY OF KETAMINE

### 5.1 Efficacy of ketamine

Ketamine is essentially a noncompetitive NMDARs antagonist that blocks open channel pores at phencyclidine binding regions, thereby stopping cation (mainly calcium) flow and thwarting neuron excitation/depolarization. Multiple RCTs investigating the subanesthetic ketamine dose (40‐min infusion of 0.5 mg/kg) have been conducted in MDD/TRD cases.^193^, ^196^ Compared with placebo, this dose led to antidepressant action in TRD of a bipolar nature typically on mood‐stabilizer therapies with no exacerbated affective switching onto hypo/mania.^195^ Ketamine was also found to rapidly alleviate suicidal thoughts.^234^, ^235^ Since few experimentally validated therapies with rapid response exist for suicide risk, ketamine represents a putative novel antidepressant drug with fast‐acting efficacy in this respect, particularly for emergency/acute cases of this nature. Consequently, subanesthetic infusion‐based dosage regimens for ketamine provided proof‐of‐concept effectiveness and a good safety/tolerability profile within small studies.^197^, ^236^ Ketamine's antidepressant functions were clinically successful; in one study, 33% of TRD cases achieved remission and approximately 50–75% of such cases exhibited alleviation of clinical symptoms following one initial dose, with even better results obtained following multiple‐dose treatment regimens.^237^ Non‐i.v.‐based routes of administration were also investigated (intramuscular/subcutaneous/oral/sublingual/intranasal), and a wide range of effectiveness levels were found, usually with reduced adverse events compared to i.v. infusions. In addition, certain ketamine metabolites have also been linked to antidepressant responses.^238^ Before the FDA approval of esketamine, its safety faced challenges due to ketamine's regulation of opioid receptors,^239^, ^240^ which raised concerns about its potential for abuse.^241^ A recent report^242^ revealed that acute opioid receptor antagonism by naltrexone decreased the antidepressant action of ketamine, which may indicate that long‐term use of ketamine for depression could lead to abuse problems. Interestingly, chronic naltrexone pretreatment did not diminish the ketamine's antidepressant effects and showed good tolerance in five patients with MDD recruited for the study.^243^ Furthermore, chronic concurrent use of buprenorphine, methadone, or naltrexone did not inhibit antidepressant activity.^244^ These divergent results can be explained by long‐term versus acute opioid blocker administration. The binding affinity of ketamine for opioid receptors and the role and precise mechanism of opiates in the antidepressant effects of ketamine should be further investigated.

### 5.2 Safety of ketamine

The opioid receptor blocker naltrexone may block the constitutive inhibition of cAMP by opioid receptors, and cAMP‐mediated neuronal nitric oxide synthase (nNOS) activation may be involved in the downregulation of mammalian target of rapamycin (mTOR) signaling, which plays a critical role in the rapid‐acting antidepressant effects of ketamine.^245^, ^246^ A series of studies have confirmed the safety and effectiveness of ketamine, and its use as an antidepressant has been globally recognized. In December 2019, the European Commission approved esketamine for patients with MDD and failed antidepressant treatment with at least two drugs,^217^, ^247^ despite doubts regarding its effectiveness,^248^, ^249^ which was ultimately determined in three acute‐phase studies and two maintenance‐phase studies. One phase 3 study of 200 cases treated with esketamine as adjunct therapy with another antidepressant demonstrated major mood‐lifting at 4 weeks in comparison to placebo.^233^ However, these studies did not reach the targeted therapeutic endpoints.^215^ Notably, studies of acute cases treated with esketamine involved patients with more severe depressive conditions than those indicated by FDA approval for antidepressant treatment with adjunctive medications (https://fda.gov/downloads/AdvisoryCommittees/CommitteesMettingMaterials/Drugs/PsychopharmacologicDrugsAdvisoryCommittee/UCM630970.pdf(2019). Two maintenance investigations monitored patients treated with esketamine on a weekly/biweekly dosage regimen for 12 months,^203^ and contributed positive datasets. Presently, Janssen (New Blueswick, New Jersey, USA) is conducting additional clinical trials to evaluate esketamine's safety profile for 5‐year treatment regimens.^250^ Esketamine presently requires administration in tandem with risk evaluation and mitigation strategy guidelines because of its previously noted fleeting dissociative/psychotomimetic adverse events risk and possible abuse/misuse when administered at antidepressant doses. Notably, (*R*)‐ketamine has also been indicated for rapid antidepressant actions, with an increased tolerance profile compared to esketamine.^231^ The latest research reports indicate that (*R*)‐ketamine generates long‐term antidepressant function with none of the adverse effects caused by (*S*)‐ketamine.^222^, ^251^ Perception pharmaceuticals have been conducting a phase I investigation on this drug since 2019, although outcome data are still pending.^231^ Even though (*R*)‐ketamine is viewed as a potentially effective treatment for TRD, no clinical trials evaluating the efficacy and safety have been done to date, which highlights the need for further research.

## 6 KETAMINE ADVERSE‐EFFECT PROFILE

A single infusion dose of ketamine is typically well tolerated, although it can induce temporary adverse events during the initial hours post‐first dosing,^252^ the most common being visual disturbances, dysphoria, dissociation, anxiety, and euphoria. Other side effects include nausea, vomiting, dizziness, drowsiness, hypertension, and tachycardia.^231^, ^253^, ^254^, ^255^ Due to the short half‐life of ketamine, such adverse events fade within minutes following discontinuation of transfusion dosing, resulting in total remission within 120 min.^256^ Swainson et al. described a set of adverse effects linked to the intranasal administration of esketamine.^250^ Ketamine/esketamine side effects that have emerged during the treatment of MDD can be classified as psychiatric (dissociation/psychotomimetic), neurologic/cognitive, hemodynamic, genitourinary, and leading to abuse risks, even from a single dose of ketamine, with cumulative effects following multiple doses, although not well investigated.^257^ Severe physical adverse events included sedation, dizziness, light‐headedness, nausea, poor coordination, vomiting, and headache, which were mostly self‐limited. Adverse psychiatric events typically occur in an acute manner (irritability, agitation, anxiety, and mood elevation) and are typically short‐lived (dissociation, disorganized thought, altered perceptions/hallucinations/illusions, emotional withdrawal, and suspiciousness), and their severity is affected by the dose, dosage regimen, and administration route. Cognitive impairment traits were also noted.^257^

Although this drug can be effective in MDD, it is essential for physicians to be aware that such adverse events can and will occur.^258^, ^259^ Furthermore, ketamine is called ‘‘Special K’’ in the narcotics‐consuming population due to its popularity as a powerful recreational drug of abuse,^260^ typically leading to temporary cognitive impairment,^185^ although long‐term abuse leads to neurotoxicity.^261^, ^262^ Prolonged ketamine abuse at excessive doses can cause urological manifestations and exacerbate the severity of adverse effects. The risk of chronic abuse of ketamine as a recreational narcotic leading to cognition/affective impairments that include depressive conditions remains a major issue of concern, and consequently raises doubts about the suitability of ketamine for treating MDD on a prolonged basis.^263^ Recognition of the mechanism for ketamine‐directed antidepressant functions could aid in the development of novel fast‐operating drugs with reduced adverse effects.

Dissociation, psychoses, and cognitive adverse effects tend to be susceptible to ketamine enantiomer presence, although no proper comparative analyses have been conducted to assess this.^231^, ^264^ Murine receptor investigations revealed that (*R*)‐ketamine rapidly induced antidepressant function, presented an improved adverse event profile compared to esketamine, and enhanced phencyclidine‐driven cognitive impairment side effects within murines.^222^, ^226^, ^264^ Such adverse effects could intensify depending on the route of administration and dosage regimen. Additional side effects associated with ketamine include neurotoxicity, bladder toxicity, and tolerance to prolonged exposure to ketamine‐based infusions.^265^ The development of ketamine metabolites as antidepressants to avoid these adverse effects is an ongoing research strategy. (*S*)‐Ketamine has been approved in the United States and Europe because of fewer side effects; Nevertheless, some concerns remain about its efficacy and side effects. (*S*)‐ketamine has a greater affinity for NMDARs than (*R*)‐ketamine and this may contribute significantly to its clinical activity, especially when given orally.^266^ Surprisingly, increasing preclinical and clinical evidence suggests that (*R*)‐ketamine may be more effective at treating depression with less side effects than (*S*)‐ketamine.^222^, ^251^, ^267^, ^268^, ^269^, ^270^ Pharmacological studies are needed to investigate the specific cellular and molecular mechanisms underlying the antidepressant effects and side effects of ketamine and its metabolites to discover fast‐acting antidepressants without undesirable side effects.

## 7 PHARMACOLOGICAL PROFILE OF KETAMINE AND ITS UNDERLYING MECHANISM

**FIGURE 3:**
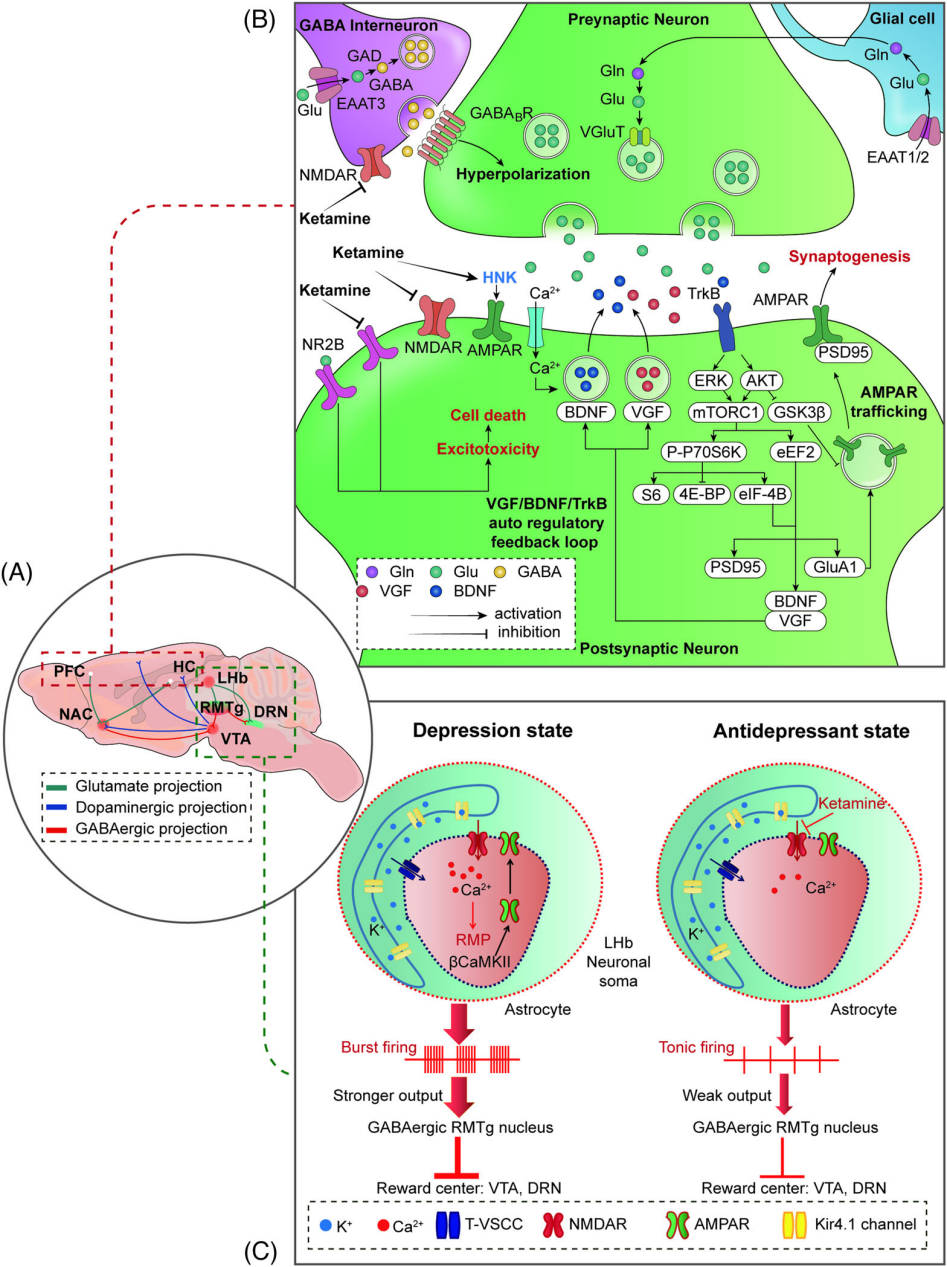
Ketamine pharmacological profile and its underlying mechanisms for rapid‐acting antidepressant action. (A) The neurocircuits implicated in the ketamine rapid antidepressant action. (B) Four potential mechanisms underlying the rapid and sustained antidepressant action of ketamine in the PFC and hippocampus: (1) Disinhibition of glutamate release from GABAergic interneurons by blocking the presynaptic NMDARs in the mPFC and hippocampus. (2) Inhibition of the extra‐synaptic NMDARs subunit (NR2B) of the pyramidal neurons in the cortex. (3) Inhibition of the spontaneous NMDARs‐mediated miniature excitatory postsynaptic current (mEPSCs) at rest in the PFC and hippocampus. (4) Direct AMPARs triggering. (C) Inhibition of NMDAR‐dependent neuron burst firing in the LHb.

Intense focus has been placed on understanding the pharmacology behind the antidepressant actions of ketamine, mainly through in vivo investigations that unravel novel mechanisms (as shown in Figure 3). Ketamine's direct targets are NMDARs that are expressed all over the brain and crucial to brain function. Ketamine produces antidepressant actions by affecting the critical brain's reward and mood circuitry regions, specifically involving the PFC, hippocampus, nucleus accumbens (NAc), ventral tegmental area (VTA), and lateral habenula (LHb).^246^, ^271^, ^272^, ^273^, ^274^, ^275^, ^276^, ^277^, ^278^, ^279^, ^280^, ^281^, ^282^ Postmortem studies have demonstrated that the PFC and hippocampal circuitry are dysregulated in depression, including alterations in structure, markers of glutamatergic and GABAergic neurotransmission, and connectivity with downstream structures,^44^ as well as reduced synaptic markers and number of synapses in the PFC and hippocampus.^282^ In addition, a decrease in prefrontal and hippocampal volumes has been demonstrated, which correlated with the length of illness.^283^

Molecular biology, morphology, anatomy, electrophysiology, and pathophysiology^284^, ^285^, ^286^, ^287^, ^288^ strongly implicate the hippocampus‐PFC circuit in MDD, which is a central hub regulated by the amygdala (glutamatergic projection) and dorsal raphe nucleus (DRN) (serotonergic projection). In contrast to the changes in the hippocampus and PFC in MDD, the volume, neuroplasticity, and neurotrophic factors in the NAc were increased,^273^, ^289^, ^290^ supporting the hypothesis that stress‐induced NAc hypertrophy may be associated with the pathophysiology of MDD. Furthermore, rewarding stimuli perceived in the hippocampus and PFC are relayed by excitatory glutamate projections to the NAc; however, the regulation of the NAc is complicated. In addition, the mesolimbic DA system is related to the MDD pathophysiology, and dopaminergic projections originate in the VTA and project to the PFC and NAc.^291^ Moreover, the LHb, as the “small” region, may have “big” driving effects in the psychiatry disorders phenotype.^292^, ^293^, ^294^, ^295^ More and more evidence shows that the ‘‘anti‐reward center’’ LHb is implicated in the coding of negative emotions^296^, ^297^, ^298^, ^299^ and crucial for the treatment and pathophysiology of MDD.^300^, ^301^, ^302^, ^303^ Adverse stimuli activate excitatory glutamate neurons projecting from the LHb to the VTA, resulting in a decrease in DA output from the VTA and a decrease in reward. Hu et al. demonstrated that high‐frequency burst firing of LHb neurons may drive depression‐like behaviors in rodents^276^, ^277^, ^278^, ^279^, ^280^, ^281^, ^282^, ^283^, ^284^, ^285^, ^286^, ^287^, ^288^, ^289^, ^290^, ^291^, ^292^, ^293^, ^294^, ^295^, ^296^, ^297^, ^298^, ^299^, ^300^, ^301^, ^302^, ^303^, ^304^ indicating an essential role for the LHb in the pathophysiology of MDD. Recently, it was shown that the low‐voltage‐sensitive T‐type calcium channel (T‐VSCC) blocker ethosuximide did not demonstrate rapid and sustained antidepressant effects in a CSDS model.^305^ A subsequent study reported that potassium channel Kir4.1 inhibitors such as quinacrine and sertraline had no rapid and sustained antidepressant effects in a CSDS model. Hence, Kir4.1 channel inhibitors are unlikely to exert robust antidepressant effects similar to ketamine although further study needs to be conducted by using selective and potent Kir4.1 channel inhibitors.^306^ Based on their different roles in the regulation of MDD, the underlying mechanisms of these brain regions in mediating the rapid and sustained antidepressant effects of ketamine have been explored recently. As shown in Table 3, multiple preclinical studies demonstrated that ketamine and/or its metabolites induce behavioral effects that predict antidepressant effectiveness.

**TABLE 3:** The preclinical study for the antidepressant effects of ketamine and metabolites

Species	Drug	Depression model	Administration paradigm	Route of administration	Timing	system	Effect	Reference
Mouse	(*2R,6R*)‐HNK	Chronic social defeat stress model	i.p.	10 mg/kg	1 h, 24 h posttreatment	Mouse hippocampus	Increased BDNF levels, decreased eEF2 phosphorylation	^226^
/	(*2R,6R*)‐HNK	/	/	50 μM	30 min exposure	Mouse primary neurons	Decreased eEF2 phosphorylation	^307^
Mouse	(*2R,6R*)‐HNK	/	i.p.	30 mg/kg 10 and 50 nM	30 min posttreatment 1 h exposure	Mouse prefrontal cortex, rat primary neurons	Increased BDNF release, Increased p‐mTOR, p‐ERK	^308^
Mouse	Ketamine	The learned helplessness (LH) model	i.p.	10 mg/kg	2–72 h posttreatment	mPFC	Enhanced glutamate‐evoked dendritic spinogenesis	^278^
Adult male mice	Ketamine (*2R,6R*)‐HNK	/	i.p.	10,30 mg/kg 20 mg/kg	1, 24, and 144 h after posttreatment 1 h after exposure	Mouse primary neurons	induced hippocampal synaptic plasticity depends on 4E‐BPs	^225^
Transgenic mice and C57BL/6J mice	Ketamine	Chronic CORT exposure	i.p.	10 mg/kg	24 h after exposure	Mouse mPFC	restoring lost spines and rescuing coordinated ensemble activity in PFC microcircuits	^277^
Mouse	(*S*)‐Ketamine	CSDS model	i.p.	10 mg/kg	7 days after injection	Mouse prefrontal cortex and hippocampus	induced dendritic spine density and synaptogenesis	^309^
C57BL/6 mice Male cLH rats	Ketamine	CRS depression model	i.p.	10 mg/kg	1 h after drug delivery	LHb	blocked bursting in the lateral habenula	^276^
Male C57BL/6 mice	Ketamine (2R,6R)‐HNK	/	i.p.	3 mg/kg 10 mg/kg	1 day after injection	NAc	Impaired Long‐term potentiation (LTP) in the NAc	^310^
C57BL/6 mice	Ketamine	/	i.p.	3 mg/kg	30 min, 3 h, and 24 h after posttreatment	hippocampus	reduced the level of eEF2 phosphorylation and strengthened synaptic responses	^311^
Sprague‐Dawley rats	Ketamine	/	i.p.	10 mg/kg	1 day after posttreatment	mPFC	VEGF signaling mediated the rapid antidepressant actions of ketamine	^279^
Adult male Sprague‐Dawley rats	Ketamine	/	i.p.	10 mg/kg	30 min or 1 week after posttreatment	mPFC Hippocampus	activated of the vHipp–mPFC pathway	^280^
Rats	ketamine	chronic stress rats	i.p.	100 nM 10 mg/kg	30 min after ketamine treatment	Hippocampal	Induced HDAC5 phosphorylation and nuclear export in Hippocampal Neurons	^281^
Rats	ketamine	/	i.p.	10 mg/kg	30 min, 1 h, 2 h, and 6 h after posttreatment	The prefrontal cortex	Activated the mTOR pathway	^246^

BDNF, brain derived neurotrophic factor; CSDS, chronic social defeat stress; eEF2, eukaryotic elongation factor 2; ERK, extracellular signal‐regulated kinase; HDAC5, histone deacetylase 5; LHb, lateral habenula; mTOR, mammalian target of rapamycin; mPFC, medial prefrontal cortex; NAc, nucleus accumbens.

Based on the above‐mentioned information, we summarized the molecular mechanisms behind ketamine's antidepressant effects as follows: (1) inhibition of glutamate release by blocking presynaptic NMDARs of GABAergic interneurons in the mPFC and hippocampus;^312^, ^313^, ^314^ (2) inhibition of extra‐synaptic NMDAR subunits (NR2B) in cortical pyramidal neurons;^315^, ^316^ (3) inhibition of spontaneous NMDAR‐mediated miniature excitatory postsynaptic currents (mEPSCs) in the PFC and hippocampus;^311^, ^317^ and (4) triggering of prosynaptogenic AMPARs.^226^, ^318^ These four hypotheses mainly focus on the dependence or independence of NMDARs, and subsequent AMPAR activation is suggested to play a role in the rapid antidepressant actions of ketamine. Ketamine activates synaptogenic intracellular signaling, including the rapid release of neurosecretory protein VGF (nonacronymic) and BDNF and subsequent activation of tropomyosin receptor kinase B (TrkB)‐mediated AKT and/or extracellular signal‐regulated kinase (ERK)/mTORC1 signaling, thereby enhancing the VGF/BDNF/TrkB autoregulatory feedback loop in the rapid and sustained antidepressant effects of ketamine. (5) Inhibition of NMDAR‐dependent burst firing of neurons in the LHb. The finding that ketamine blocks burst firing in the LHb to rapidly relieve depression confirms that the LHb may be the trigger subregion in the rapid‐acting antidepressant‐like effects of ketamine. (6)Transforming growth factor β1(TGF‐β1) system. TGF‐β1 in microglia may be linked to the antidepressant properties of (*R*)‐ketamine in animal models of depression.^319^

### 7.1 Underlying molecular signaling in the rapid and sustained antidepressant action of ketamine

Imbalance of inhibitory and excitatory neurotransmission in the PFC and hippocampus has been involved in depression and convergent evidence from clinical and preclinical studies indicates that dysfunction of glutamatergic and GABAergic systems may contribute the pathophysiology of MDD.^44^, ^56^ MDD is generally accompanied by low GABA levels or GABAergic interneuron numbers, possibly disinhibiting glutamate release.^320^ Ketamine was reported to inhibit presynaptic NMDARs of GABAergic interneurons present in excitatory neurons within the hippocampus and/or mPFC regions, resulting in the release of tonic inhibition that subsequently leads to increased firing of pyramidal neurons, augmenting synaptic transmission, thus orchestrating quick‐acting antidepressant functions.^44^, ^313^, ^314^ Importantly, the burst of glutamate is thought to occur via blockade of NMDARs on GABA interneurons, which are more sensitive to the open‐channel blocking actions of ketamine.^314^ This mechanism reveals that downstream AMPAR activation is dependent on ketamine‐induced presynaptic‐mediated glutamate release. Enhanced AMPARs triggering, together with ketamine‐based blockade of extra‐synaptic NMDARs, begins and aids postsynaptic triggering of neuroplasticity‐linked AMPARs subunit expression and synaptic intensity, together with synaptogenesis.^310^, ^321^, ^322^

Many rapid antidepressants, including ketamine, produce postsynaptic membrane depolarization that initiates intracellular secondary signaling transduction cascades, leading to the enhanced BDNF and VGF rapid release^273^, ^323^ and subsequent activation of TrkB‐mediated AKT and/or ERK/mTORC1 signaling,^309^, ^324^ indicating that TrkB may be the key regulator underlying ketamine‐induced rapid antidepressant actions. More recent findings confirm that TrkB is required for ketamine‐induced synaptic potentiation^325^ and TrkB activation‐induced allosteric facilitation of BDNF signaling is a common mechanism for rapid antidepressant actions.^326^ Additionally, TrkB‐dependent adult hippocampal progenitor differentiation mediates a sustained ketamine antidepressant response.^327^ It is worth noting that the production of BDNF and VGF enhances the VGF/BDNF/TrkB autoregulatory feedback loop in ketamine's rapid‐acting and sustained antidepressant efficacy.^323^ The production of VGF and BDNF may also be regulated by mTORC1; notably, downregulation of mTORC1 signaling pathway has been identified in postmortem tissue from individuals with MDD^328^ and directly targeting mTORC1 produces rapid antidepressant action,^329^ which supports the idea that mTORC1 is the common downstream kinase involved in the rapid‐acting antidepressant effects of ketamine and (*2R,6R*)‐HNK in rodents.^246^, ^308^ mTORC1 controls various neuronal functions, particularly through eukaryotic initiation factor 4E‐binding proteins (4E‐BPs) and eukaryotic elongation factor 2 kinase (eEF2K), which regulate protein synthesis through downstream effects. A more recent report demonstrated that brain 4E‐BPs and downstream initiation of mRNA translation are pivotal targets of ketamine and (*2R,6R*)‐HNK.^225^ Preclinical studies have also identified eEF2K signaling as essential for the rapid antidepressant action of ketamine.^307^, ^330^, ^331^ Ketamine deactivates eEF2K and thereby decreases the amount of phosphorylated eEF2K, eliciting desuppression of dendritic protein translation, ultimately triggering synaptic upscaling.^311^, ^332^, ^333^

In addition to the regulation of presynaptic NMDARs, postsynaptic NMDARs also play a vital role in the antidepressant actions of ketamine. In particular, NR2B‐containing heterotetramers, the primary subunits of NMDARs, are mainly activated by ambient glutamate^334^ and mediate synaptic homeostasis via suppression of protein synthesis.^335^ Thus, antagonism of the NR2B subtype may be a promising target for developing novel antidepressants with more powerful effects and quicker onset compared with traditional antidepressants.^336^ Ketamine also suppresses protein synthesis and produces rapid antidepressant actions through the extrasynaptic NR2B‐dependent mechanism.^315^, ^316^ Ketamine appears to exert its antidepressant effects by blocking NMDARs‐mediated miniature excitatory postsynaptic currents at rest, leading to deactivation of the calcium‐/calmodulin‐dependent kinase eEF2K, resulting in dephosphorylation of eEF2 and subsequent desuppression of BDNF, VGF, and AMPARs subunit GluA1 protein translation.^323^, ^337^, ^338^ Hypofunction of the midbrain reward center has been reported in depression.^339^ Recently, the reward centers (including the VTA and DRN) were found to be inhibited by the LHb dependent on the GABAergic rostromedial tegmental (RMTg) nucleus,^340^, ^341^ indicating that the LHb has thought to be an important brain region in the pathophysiology of depression.

LHb neurons were previously classified as silent, tonic firing, and burst firing types,^342^, ^343^ and an increase in burst firing neurons and spikes in burst mode in the LHb was characterized as the novel pathogenesis of MDD. Preclinical and clinical studies have revealed that the LHb is metabolically hyperactive,^344^, ^345^ and alleviation of burst activity in the LHb may be sufficient to prevent depressive‐like symptoms. Because LHb bursts depend on NMDARs, the rapid antidepressant pharmacological actions and unique mechanisms of ketamine in the LHb have caused wide public concern. A pair of studied from Hailan Hu's laboratory^276^, ^304^ demonstrated that ketamine blocks the burst firing of neurons in the LHb in an NMDARs‐dependent manner. The potential mechanisms indicate that ketamine can quickly alleviate symptoms of depression by disinhibiting reward centers through blocking LHb bursts. This striking finding might explain the mechanism of LHb bursts and provide new insights into the development of novel antidepressant targets in the LHb.

### 7.2 Convergent onset target: AMPARs and trafficking regulation

Ketamine is a full antagonist of NMDARs, and its structural basis on human NMDARs has been described by cryoelectron microscope.^346^ However, whether the rapid‐acting antidepressant actions of ketamine and its metabolites relay on NMDARs have been questioned. Unlike ketamine, other NMDARs antagonists (lanicemine, memantine, and N_2_O) do not show significant antidepressant properties,^347^ indicating that other additional mechanism may involve in the antidepressant regulation of ketamine. Notably, recent finding of NMDARs suppression‐independent antidepressant actions of ketamine metabolites^226^ indicate a novel mechanism underlying ketamine's unique antidepressant actions; therefore, future studies should not be limited to NMDARs antagonists, because this suggests that ketamine's mechanisms for rapid‐acting antidepressant‐like effects is complicated. Additional targets within the glutamatergic system include inotropic and metabotropic receptors and glutamate transporters. The affinity of (S)‐ketamine for NMDARs is approximately fourfold greater than (*R*)‐ketamine,^348^ and may explain the greater potential anesthetic effects and greater undesirable psychotomimetic side effects than (*R*)‐ketamine. However, (*R*)‐ketamine showed greater potency and longer term antidepressant actions than (*S*)‐ketamine,^223^ indicating that the anesthetic and psychotomimetic actions of ketamine are mediated primarily by the blockade of NMDARs. Importantly, except to blocking NMDARs, the rapid and long‐lasting antidepressant actions of ketamine and/or (*2S,6S;2R,6R*)‐HNK are also dependent on the activation of AMPARs.^226^ In addition, synaptic plasticity changes involving AMPARs are thought to underlie the long‐term antidepressant actions of ketamine.^72^, ^349^ The AMPARs‐based long‐term antidepressant function of (2*R*,6*R*)‐ HNK through the exclusion of intense bonding attractiveness for NMDARs^226^ has been found, which implicate underlying AMPARs‐mediated maintenance of synaptic potentiation in the sustained antidepressant effects. The expression, distribution, and trafficking of AMPARs play a critical role in mediating the majority of fast excitatory synaptic transmissions and neuropsychiatry disorders. The metabolite of ketamine seems to be linked to intense immediate expansion in excitatory neurotransmission through AMPARs triggering, continued through sustained maintenance by upregulation of GluA1 and GluA2 AMPARs synaptic subunits.^226^, ^350^ Convergent evidence supports the hypothesis that AMPARs‐triggering/mTOR/BDNF/VGF signaling orchestrates ketamine‐driven synaptogenesis and antidepressant function.^42^, ^351^, ^352^

By increasing BDNF and VGF release, ketamine upregulates the surface AMPARs subunits expression^353^ required for the increase in synaptic efficacy and the antidepressant effects.^354^, ^355^ In addition to AMPARs‐mediated BDNF and VGF release, AMPARs‐mediated 5‐HT release may also be involved in the rapid antidepressant‐like actions of ketamine. Ketamine can increase the levels of extracellular 5‐HT in the mPFC via AMPAR activation,^356^, ^357^ and the antidepressant‐like actions of ketamine are blocked by pretreatment with a 5‐HT‐depleting agent^358^, ^359^ and 5‐HT1A receptor antagonist.^360^, ^361^ In contrast, selective stimulation of 5‐HT1A receptors in the mPFC exerts rapid and sustained antidepressant‐like effects via activation of AMPAR/BDNF/mTOR signaling in mice, which provides evidence for the targeting of 5‐HT1A receptor in the treatment of MDD.^362^ However, pretreatment with the AMPARs inhibitor NBQX does not block the antidepressant actions of monoamine‐based antidepressants.^352^, ^363^ AMPAR is the specific target of current findings of rapid antidepressants (e.g., ketamine, GluN2B‐NMDARs antagonists, 4‐chlorokynurenic acid, GLYX‐13, scopolamine, mGluR2/3 antagonists, GABAAR‐NAMs, and (2*R*,6*R*)‐HNK).^360^, ^364^, ^365^ In support of this hypothesis, AMPARs‐positive allosteric modulators have been found to induce antidepressant‐like responses in rodents,^366^, ^367^, ^368^ which further makes AMPARs a promising target for the development of new antidepressant drug.

Previous studies have found that the AMPARs trafficking regulated by glycogen synthase kinase‐3 (GSK3)^369^, ^370^ and numerous associates between GSK3 and depression have been reviewed,^371^ which suggest that abnormally active GSK3 contributes to susceptibility to depression and inhibition of GSK3 may as one potential downstream target of ketamine in the antidepressant process. Ketamine treatment in rodents has been reported to inhibit cerebral glycogen synthase kinase‐3β(GSK‐3β), a pharmacological pathway shared by lithium.^43^, ^369^, ^372^ GSK‐3β also phosphorylates postsynaptic density‐95 (PSD‐95) protein, which regulates AMPARs trafficking.^373^ These interactions raise the possibility and confirmed that ketamine increases membrane AMPARs subunits by its inhibitory effect on GSK‐3β dependent on the phosphorylation of PSD‐95.^42^ Except for the direct targeting of AMPARs in rapid antidepressant actions, AMPARs trafficking is believed to underlie higher brain functions and has been involved in a large number of psychiatric disorders, including MDD.^42^, ^367^, ^374^ Because relatively little is known about these mechanisms of action, it is of significant importance to elucidate the underlying molecular mechanisms that regulate AMPARs trafficking in antidepressants.

## 8 SUGGESTED ROUTES OF ADMINISTRATION AND DOSAGE REGIMENS OF KETAMINE

A variety of administration routes are available for ketamine.^375^, ^376^ Intranasal administration is considered a more attractive option because it is less invasive, causes rapid systemic absorption in to the body, and is not affected by hepatic metabolism compared with intravenous administration. One RCT focusing on intranasal ketamine administration supported the feasibility of this route of administration;^232^ however, a similar study was cancelled due to decreased tolerability.^377^ Alternative clinical‐based studies have described that despite the experimental findings, maintenance doses of intranasal ketamine can be of clinical utility in cases with no other therapeutic possibilities.^378^, ^379^ Intranasal esketamine resolves several adverse effect challenges, allowing it to be approved by multiple regulatory bodies on a global scale. However, the issue of reduced effectiveness remains.^380^ Investigations on sublingual ketamine were described in a newly published review article,^381^ although such investigations had large dose‐range variations and did not consider the decreased bioavailability present in oral drug formats, thus underestimating effectiveness.^382^

Intramuscular/subcutaneous routes could be feasible for ketamine delivery,^383^ although few investigations have been conducted. Although the main route of administration of ketamine remains i.v., it can also be delivered through subcutaneous, intramuscular, transdermal, intranasal, intrarectal, or oral routes. Bioavailability of drugs differs depending on their administration route. The bioavailability profiles are as follows: i.v. route (100%), intranasal (45%), sublingual (30%), oral (20%), intramuscular (93%), and rectal (30%). Ketamine is highly metabolically processed, with a plasma redistribution half‐life of 4 min and plasma terminal half‐life lasting 2.5 h.^256^ Risk‐benefit evaluations have increased the focus on ketamine for patients suffering from extreme depressive conditions. However, due to the lack of current RCTs/proper placebos, reduced datasets regarding long‐term outcomes, and possible risks, ketamine treatments remain limited to the hospital scenario.

Scarce information exists regarding the optimized dosage regimen, ideal route of administration, and safety profile for repeated/long‐term use of ketamine.^384^, ^385^ Reduced dosing (2 mg/kg) and other routes of administration (intramuscular/intranasal) can contribute to antidepressant functions that are equivalent to standardized 0.5 mg/kg IV doses.^232^, ^386^ Ketamine‐induced antidepressant function is extended via multiple dosing regimens.^197^, ^252^ Datasets from pilot studies indicate that a limit of 6 i.v. infusions (0.5 mg/Kg) given three times weekly across a 14‐day timespan have good tolerance profiles and extend ketamine‐induced antidepressant functions accordingly.^197^

## 9 CHALLENGES FOR USE OF KETAMINE IN MDD

Even though several psychiatrists/anesthesiologists presently administer ketamine in outpatient scenarios, major hurdles still persist regarding widespread use. Best practice standardizations need to be implemented for optimized routes of drug administration, particularly with regard to doses and dosage regimens. Multiple investigations have focused on other routes of administration, such as intranasal esketamine, although comparative analyses for various models have not been performed. Regarding dose optimization, excluding a small (*n* = 4) placebo‐regulated crossover investigation, all RCTs on TRDs and bipolar depressive disorders employed a dosage of 0.5 mg/kg. However, the dose‐response curve for ketamine‐driven antidepressant effect is currently being evaluated within a multicenter, psychoactive, placebo‐regulated, parallel‐design study using midazolam (0.045 mg/kg) and different ketamine infusion‐based doses. It must be emphasized that since ketamine's antidepressant function is fleeting, plans for maintaining responses/circumventing relapses are essential in clinical settings. One plan is the introduction of multiple doses (boosters), similar to maintenance treatments in electroconvulsive therapy. However, few investigations have focused on multiple‐infusion ketamine therapies for MDD or only described less than 10 infusions across 12–21‐day time frames. The risks of abuse/possible sustained adverse effects (such as cognitive issues or urinary cystitis) can increase with multiple dosing regimens. This requires intense monitoring and appropriate consultation.

Ketamine's antidepressant effect can be inflated through minimal responses to i.v. saline placebo. However, ketamine variations were not considerable, stemming from standardized placebo responses during MDD trials. Although midazolam is a more suitable placebo than saline, it has its own shortcomings, namely, reduced acute dissociative adverse effects, compromising trial blindness within savvy participating patients. Subsequent research hurdles include the development of improved controls compared to midazolam and formal evaluation of randomization expectations.

Another challenge is the recognition of subgroups with increased antidepressant responses to ketamine. Multiple nonredundant clinical predictive factors for ketamine‐driven antidepressant activities are known, including Body Mass Index, family history of alcohol abuse within close relatives, and dimensional anxious depression states. Apart from such variables, multiple genomic, innate neurobiological, and fringe factors have also been demonstrated to be associated with the antidepressant effectiveness of ketamine. However, very few investigations have merged such factors/datasets to enhance prediction potential and recognize minute influences. Stemming from MDD heterogeneity, such combination routes should be conducted through multicenter ketamine MDD consortiums to maximize population cohort sizes of aggregated subgroups regarding possible mechanism‐based investigations.

**FIGURE 4:**
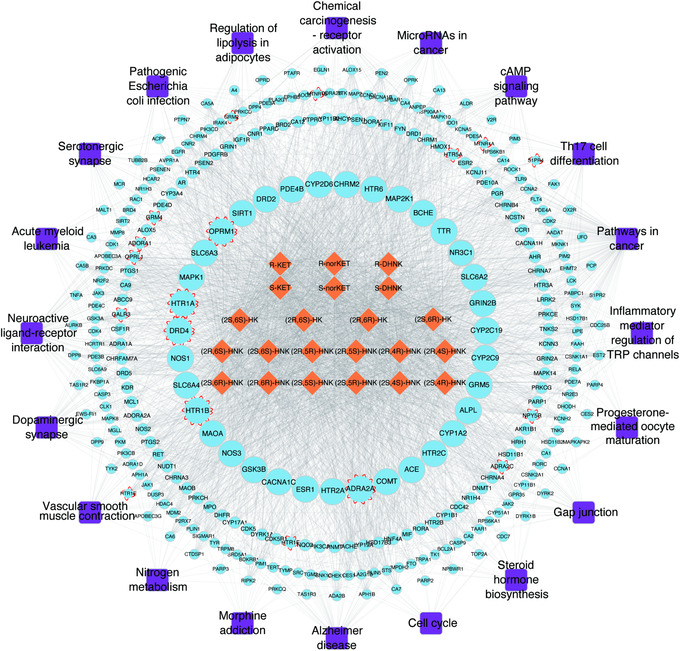
The construction and analysis of the Compound‐Target‐Pathway. The candidate ketamine metabolites (yellow diamond mesh node) are divided into the following groups based on the metabolic processes: (R,S)‐KET, (R,S)‐norKET, (R,S)‐DHNK, (2,6)‐HKs, and (2,6; 2,5; 2,4)‐HNKs. The figure was drawn by Cytoscape.

**FIGURE 5:**
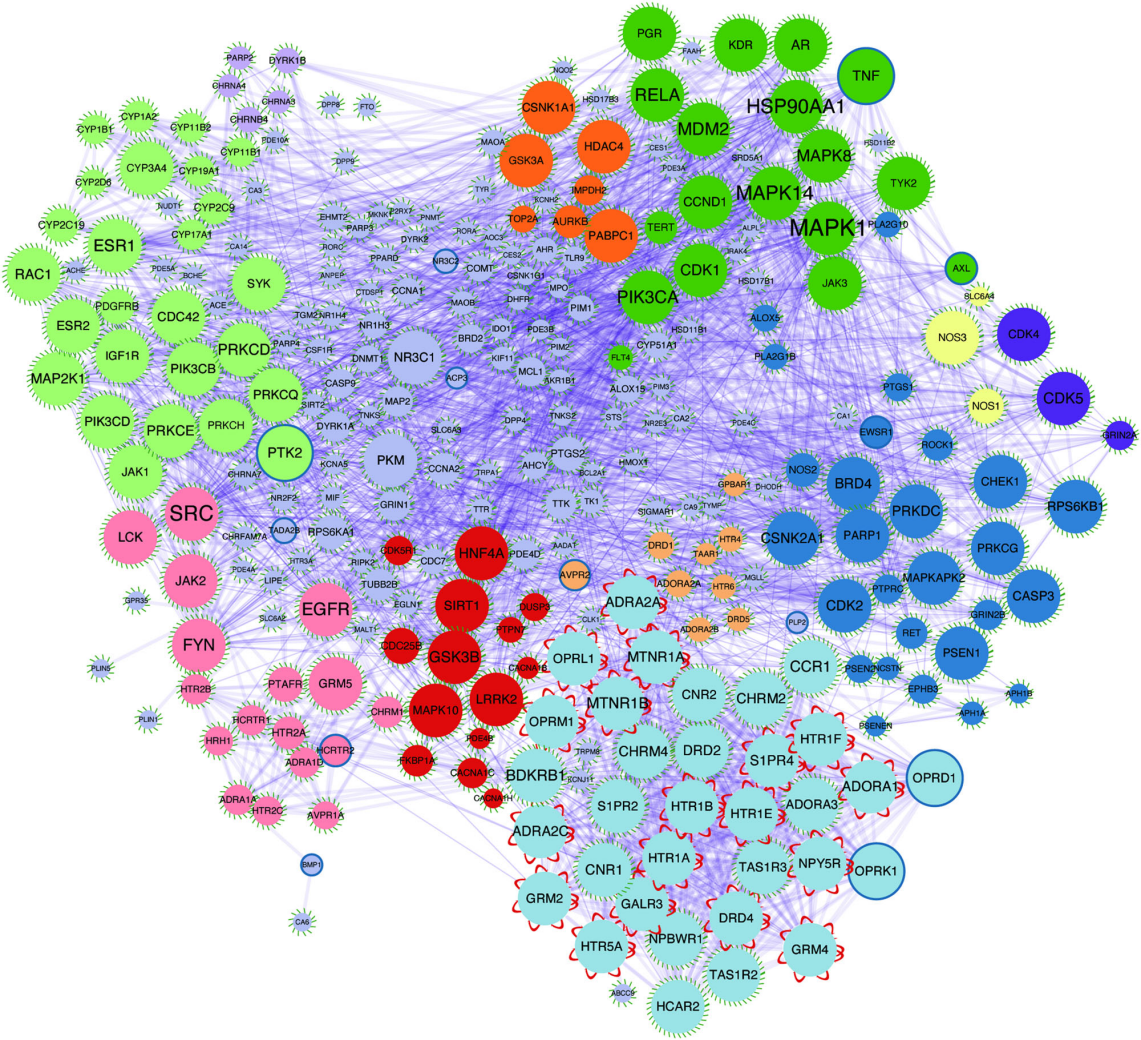
The protein–protein interaction (PPI) networks of ketamine metabolites. The figure was drawn by Cytoscape.

Additional concerns include confirmation of the sensitive/specific targets of ketamine and its metabolites. Such models could enhance the knowledge of ketamine antidepressant pharmacology, together with its use in glutamate‐dependent drug screens. In addition, the construction and analysis of the compound‐target‐pathway network and the protein–protein interaction (PPI) network of ketamine metabolites are shown in Figures 4 and 5. Many novel hubba proteins and MDD‐risk proteins were found, indicating that the current pharmacological mechanisms were just the tip of the iceberg.

Long‐term use of intravenous ketamine and intranasal esketamine was associated with an increased potential risk of addiction and suicidal incidences occurred after termination of therapy. Further studies should be conducted to explore the clinical side effects biomarkers of long‐term use of esketamine based on the technology of pharmacogenomics and clinical pharmacology. Moreover, the joint application of ketamine combined with nondrug treatment (manual‐based psychotherapies)^387^ provides a comprehensive intervention for MDD. Meanwhile, such drugs must be administered solely within institutions with the necessary infrastructure and staffing resources required for safe and reliable treatment and monitoring of the patient during their therapeutic stay.

## 10 CONCLUSION

The recently approved esketamine formulation for TRD reflects a significant advance in psychiatric therapeutics, whereby no novel mechanism‐based agents have been generated in nearly 50 years. The identification of ketamine and its derivatives has great potential for quick and effective depression‐condition therapies. However, multiple issues must be addressed. First, ketamine is a narcotic with severe adverse effects; thus, novel drugs with lower adverse event profiles are desired. Second, even though ketamine provides fast antidepressant functions, such functions only last for 7 days, leading to patient relapses. Novel drugs that can be utilized on a daily basis are highly desired. Third, investigations are needed to clarify how novel ketamine‐driven synapses wither within 7 days and whether specific mechanisms and/or drugs exist that could possibly maintain such synaptic presence, together with similar clinical functions. Fourth, more investigations are required on the cell‐based mechanism for ketamine functions, and other similar quick‐acting drugs, to determine essential pathophysiological dysfunctions leading to depressive conditions. Fifth, prolonged effect and safety profile for ketamine therapy (over 14 days), especially regarding multiple doses, requires further research.

Future research regarding ketamine should focus on crystallizing ketamine pharmacology, clarifying the administration profile that will induce prolonged therapeutic benefits, analyzing ketamine safety profiles in multiple‐/low‐dose formulations, and recognizing novel biomarkers for differentiating ketamine response within patient cohorts.

## CONFLICTS OF INTEREST

The authors disclose no potential conflicts of interest.

## ETHICS APPROVAL

Not applicable.

## AUTHOR CONTRIBUTION

Chuang Wang and Jia Xu conceived the manuscript; Haihua Tian, Zhenyu Hu, and Jia Xu wrote and edited the manuscript. All authors read and approved the final manuscript. Haihua Tian and Zhenyu Hu contributed equally to this work.

## Data Availability

The datasets in this study are available from the corresponding author on reasonable request.
